# Dual roles of Aβ in proliferative processes in an amyloidogenic model of Alzheimer’s disease

**DOI:** 10.1038/s41598-017-10353-7

**Published:** 2017-08-30

**Authors:** David Baglietto-Vargas, Elisabeth Sánchez-Mejias, Victoria Navarro, Sebastián Jimenez, Laura Trujillo-Estrada, Angela Gómez-Arboledas, Maria Sánchez-Mico, Raquel Sánchez-Varo, Marisa Vizuete, José Carlos Dávila, José Manuel García-Verdugo, Javier Vitorica, Antonia Gutierrez

**Affiliations:** 1 Dpto. Biología Celular, Genética y Fisiología, Facultad de Ciencias, Instituto de Biomedicina de Málaga (IBIMA), Universidad de Málaga, Málaga, Spain; 2 Dpto. Bioquímica y Biología Molecular, Facultad de Farmacia. Universidad de Sevilla, Sevilla, Spain; 3 Laboratorio de Neurobiología Comparada, Dpto. Biología Celular. Instituto Cavanilles de Biodiversidad y Biología Evolutiva, Universidad de Valencia, Valencia, Spain; 4 Centro de Investigación Biomédica en Red sobre Enfermedades Neurodegenerativas (CIBERNED), Madrid, Spain; 5 Instituto de Biomedicina de Sevilla (IBIS)-Hospital Universitario Virgen del Rocio/CSIC/Universidad de Sevilla, Seville, Spain; 6 Institute for Memory Impairments and Neurological Disorders (UCIMIND), University of California, Irvine, USA

## Abstract

Alzheimer’s disease is a major neurodegenerative disorder that leads to severe cognitive deficits in the elderly population. Over the past two decades, multiple studies have focused on elucidating the causative factors underlying memory defects in Alzheimer’s patients. In this regard, new evidence linking Alzheimer’s disease-related pathology and neuronal stem cells suggests that hippocampal neurogenesis impairment is an important factor underlying these cognitive deficits. However, because of conflicting results, the impact of Aβ pathology on neurogenesis/gliogenesis remains unclear. Here, we investigated the effect of Aβ on neuronal and glial proliferation by using an APP/PS1 transgenic model and *in vitro* assays. Specifically, we showed that neurogenesis is affected early in the APP/PS1 hippocampus, as evidenced by a significant decrease in the proliferative activity due to a reduced number of both radial glia-like neural stem cells (type-1 cells) and intermediate progenitor cells (type-2 cells). Moreover, we demonstrated that soluble Aβ from APP/PS1 mice impairs neuronal cell proliferation using neurosphere cultures. On the other hand, we showed that oligomeric Aβ stimulates microglial proliferation, whereas no effect was observed on astrocytes. These findings indicate that Aβ has a differential effect on hippocampal proliferative cells by inhibiting neuronal proliferation and triggering the formation of microglial cells.

## Introduction

Alzheimer’s disease (AD) currently represents one of the most prevalent neurodegenerative disorders affecting the elderly population^1^. AD is an irreversible and progressive disease that leads to a gradual loss of memory and other cognitive functions^1, 2^. Despite the intensive research over the past three decades, the mechanisms underlying cognitive and memory deficits in AD are elusive. Recent evidence indicated that hippocampal neurogenesis plays a key role in learning and memory processes, and numerous findings support the concept that neurogenesis impairment may be associated with the cognitive decline observed in AD patients^3–11^. In addition, several studies have shown that neurogenesis in the subgranular zone (SGZ) plays an important role with respect to hippocampal spatial and contextual memories, demonstrating an important link between adult neurogenesis and cognitive processes^9, 11^. Furthermore, supporting this idea, several compelling studies have shown that the inhibition of adult hippocampal neurogenesis impairs memory processing, while its enhancement improves memory performance^12–15^.

Studies involving human *post mortem* AD cases have shown contradictory results. While some reports indicated that AD is associated with a marked increase in the proliferation and survival of new neurons^16, 17^, others have shown a significant reduction in the immature neuron population at severe stages of the disease^18–20^. Interestingly, it has been reported that proliferative cells in AD brains were mainly derived from glia- and vasculature-associated changes, suggesting that the proliferating cells do not become mature neurons^18, 19, 21, 22^. Overall, these data suggest that adult hippocampal neurogenesis is differentially affected during the progression of the disease and that different cellular proliferative phases may occur.

To understand the impact of AD on neurogenesis/gliogenesis, multiple studies have been performed using several AD animal models. Overall, there is a consensus indicating that hippocampal neurogenesis is altered in amyloidogenic mouse models of AD^23–26^; however, the contradictory results observed in human cases have also been reported in these models. These conflicting results observed in AD models have been proposed to depend on mouse genetic background, gender, mutation and disease progression features^17, 26–30^. Here, we provide critical evidence for the impact of Aβ on hippocampal neuron/glia proliferation in different cell types using an APP/PS1 mouse model of AD. Our study showed that neurogenesis is impaired early on due to the remarkable diminution of SGZ progenitor cells (including type-1 and type-2 progenitors), which could be due to the accumulation of extracellular Aβ. Additionally, we showed that soluble Aβ inhibits the proliferation and growth of hippocampal neurospheres, therefore affecting the formation of new neurons. On the other hand, our study showed that Aβ stimulates microglial proliferation *in vivo* and *in vitro*. Moreover, no differences were observed in astroglial cells. Therefore, our study suggests a differential effect of Aβ on neurons and glial cells.

## Results

### Neurogenesis is impaired in the SGZ of APP/PS1 mice

**Figure 1:**
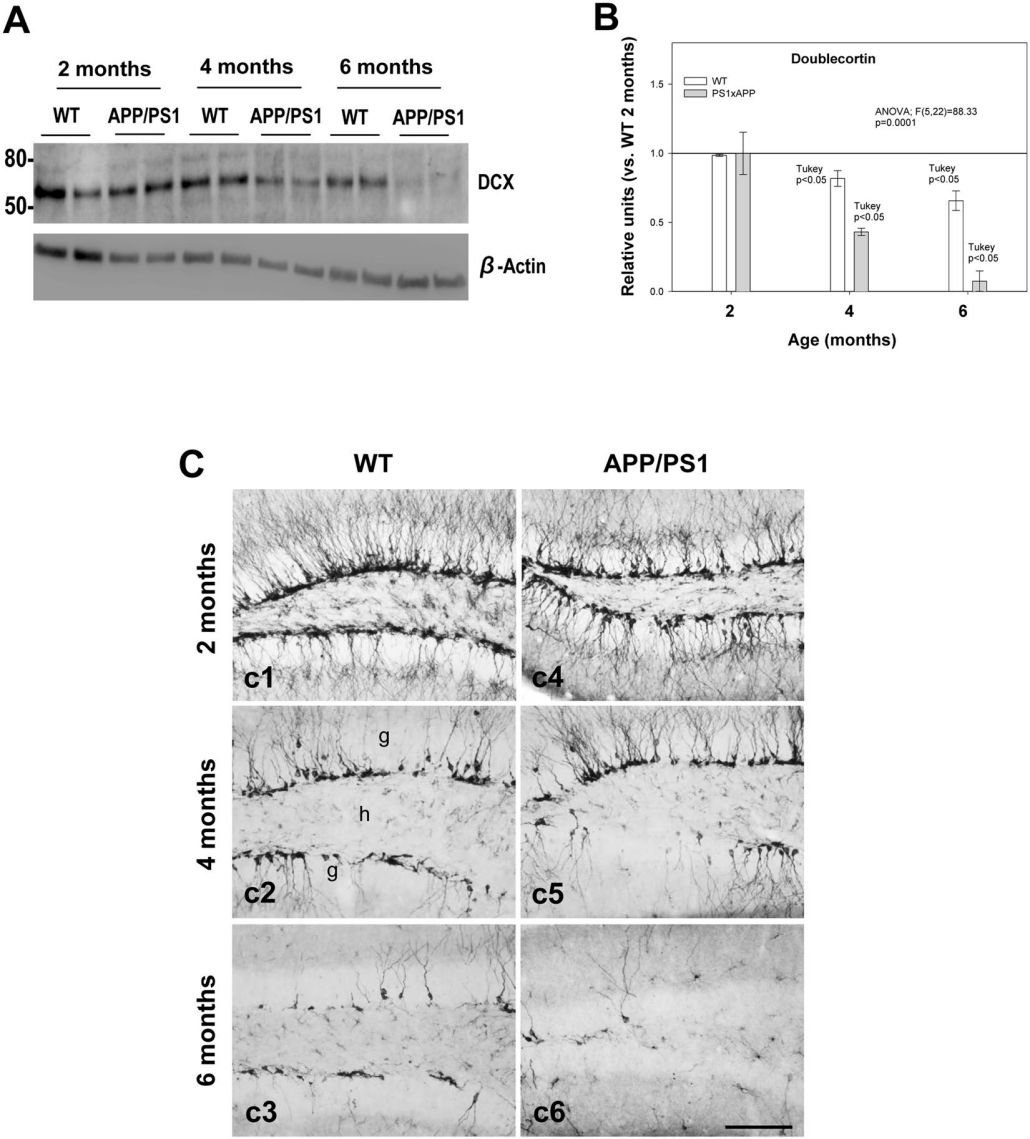
Neurogenesis is severely affected in the SGZ of APP/PS1 mice. (**A** and **B**) Western-blot analysis shows a significant decrease (n = 5/genotype/age; ANOVA F(5,22) = 88.33; Tukey’s post hoc p < 0.05) in the level of DCX at 4 and 6 months of age in APP/PS1 mice compared with age-matched WT mice. (**C**) Light microscopic images of DCX immunolabeling in the dentate gyrus of WT (c1–c3), and APP/PS1 (c4–c6) mice from 2 to 6 months of age. Significant reduction in DCX-positive cells was observed in APP/PS1 mice compared to WT mice during aging; g: granular cell layer; h: hilus. Scale bars: 100 μm (c1–c6).

To assess whether neurogenesis was affected in the subgranular zone of the dentate gyrus (DG) in APP/PS1 mice, we analyzed the expression of doublecortin (DCX), a microtubule-stabilizing factor that is expressed specifically in immature neurons^31^, at 2, 4 and 6 months of age in comparison to age-matched WT mice. Western blot analysis (Fig. 1A and B) showed a significant decrease in the steady-state levels of DCX in APP/PS1 mice at 4 months and an even more severe decrease at 6 months (−56.80 ± 2.5% and −92.5 ± 7.3%, respectively; Tukey’s post hoc test, p < 0.05). A significant age-dependent progressive reduction of DCX was also detected in WT mice (Fig. 1A and B). These data were consistent with a prominent decrease in the number of DCX-immunopositive cells in the SGZ that was observed with age in both WT and APP/PS1 mice (Fig. 1C). The reduction of DCX-positive cells in APP/PS1 compared to age-matched WT mice was noticeable at 6 months of age (Fig. 1C, c6 compared to c3). Overall, these results indicate that neurogenesis in the SGZ is impaired in APP/PS1 mice at early stages of the disease (4- to 6-month-old mice) (see Fig. 1 and Supplemental Fig. 1).

### Proliferative cells are significantly reduced in the SGZ of APP/PS1 mice

**Figure 2:**
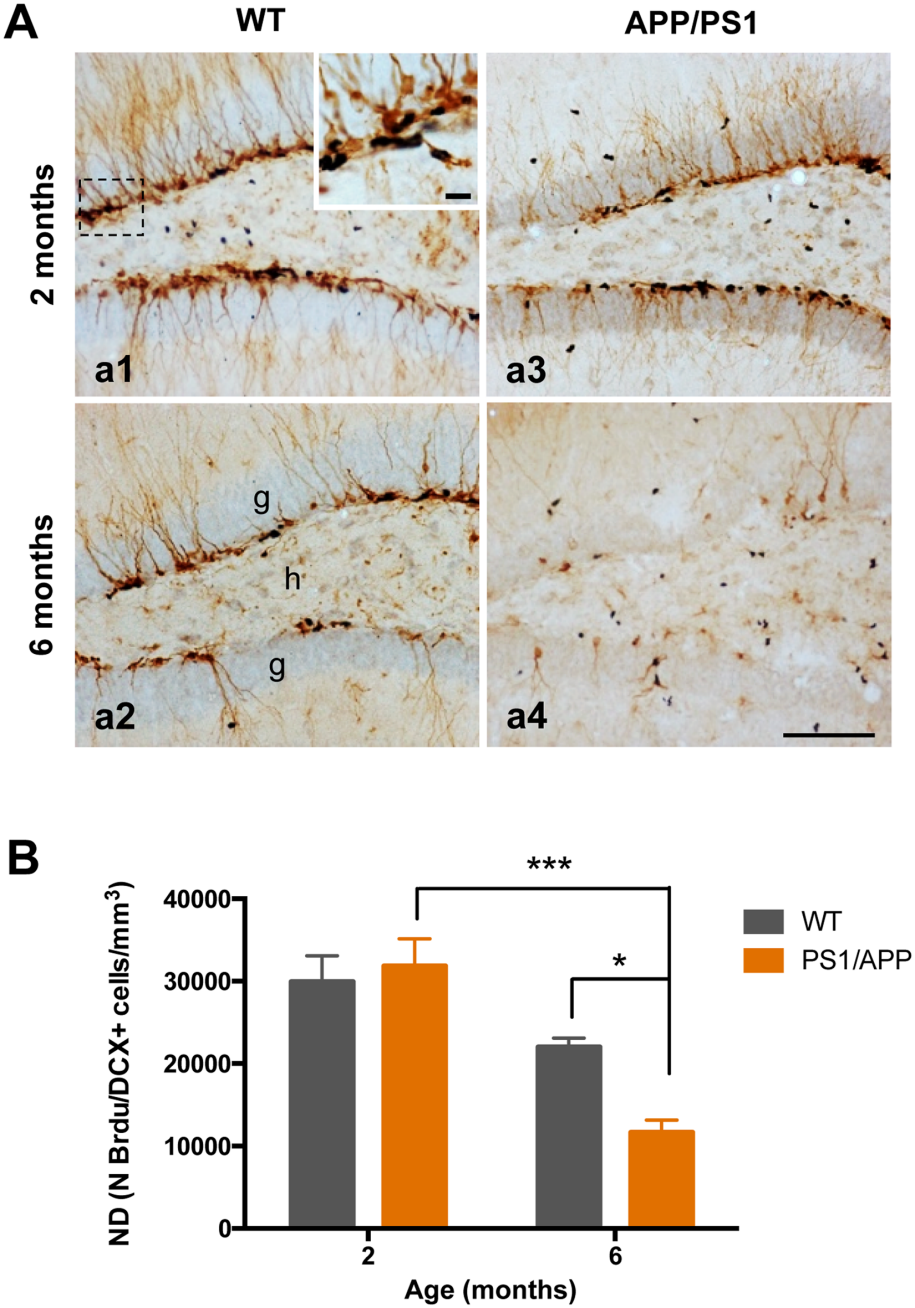
Cell proliferation is significantly reduced in the hippocampus of APP/PS1 mice at 6 months of age. (**A**) Light microscopic images of double immunolabeled cells, BrdU (blue)-DCX (brown), in the DG of 2- and 6-months-old WT (a1 and a2), and APP/PS1 (a3 and a4) mice. (**B**) The total number of BrdU/DCX-positive cells was significantly reduced in APP/PS1 mice compared to WT mice at 6 months of age (n = 4/age/genotype); ANOVA F(3,11) = 15.57; Tukey’s post hoc test *p < 0.05, ***p < 0.001. Scale bars: 100 μm (a1–a4).

To understand the mechanism underlying the severe reduction of DCX cells in the SGZ of APP/PS1 mice compared to WT mice at 6 months of age, we examined whether cell proliferation was affected in these mice. To evaluate the cell proliferation, mice were administered bromodeoxyuridine (BrdU) injections and sacrificed 3 days after the first injection (proliferation). After that, brain sections were double immunostained with anti-BrdU and anti-DCX antibodies (Fig. 2A). Stereological quantification in the SGZ showed a significant decrease in the number of double BrdU/DCX-positive cells in APP/PS1 mice at 6 months of age compared to age-matched control mice (−46.99 ± 13.45%; Tukey’s post hoc test, p < 0.05), whereas no differences between the genotypes were detected at 2 months of age (Fig. 2B). In addition, a significant reduction in the number of double-positive cells was detected in the APP/PS1 mice between 2 to 6 months of age (−63.37% ± 9.30%; Tukey’s post hoc test, p < 0.001) indicating the existence of an age-related decrease in cell proliferation (Fig. 2B). Together, these data suggest that cell proliferation is significantly diminished in the SGZ of 6-month-old APP/PS1 mice compared to age-matched controls, and the reduced cell proliferation might be responsible for the decrease in neurogenesis observed in these mice.

### Progenitor cells are extensively reduced in the SGZ of APP/PS1 mice

**Figure 3:**
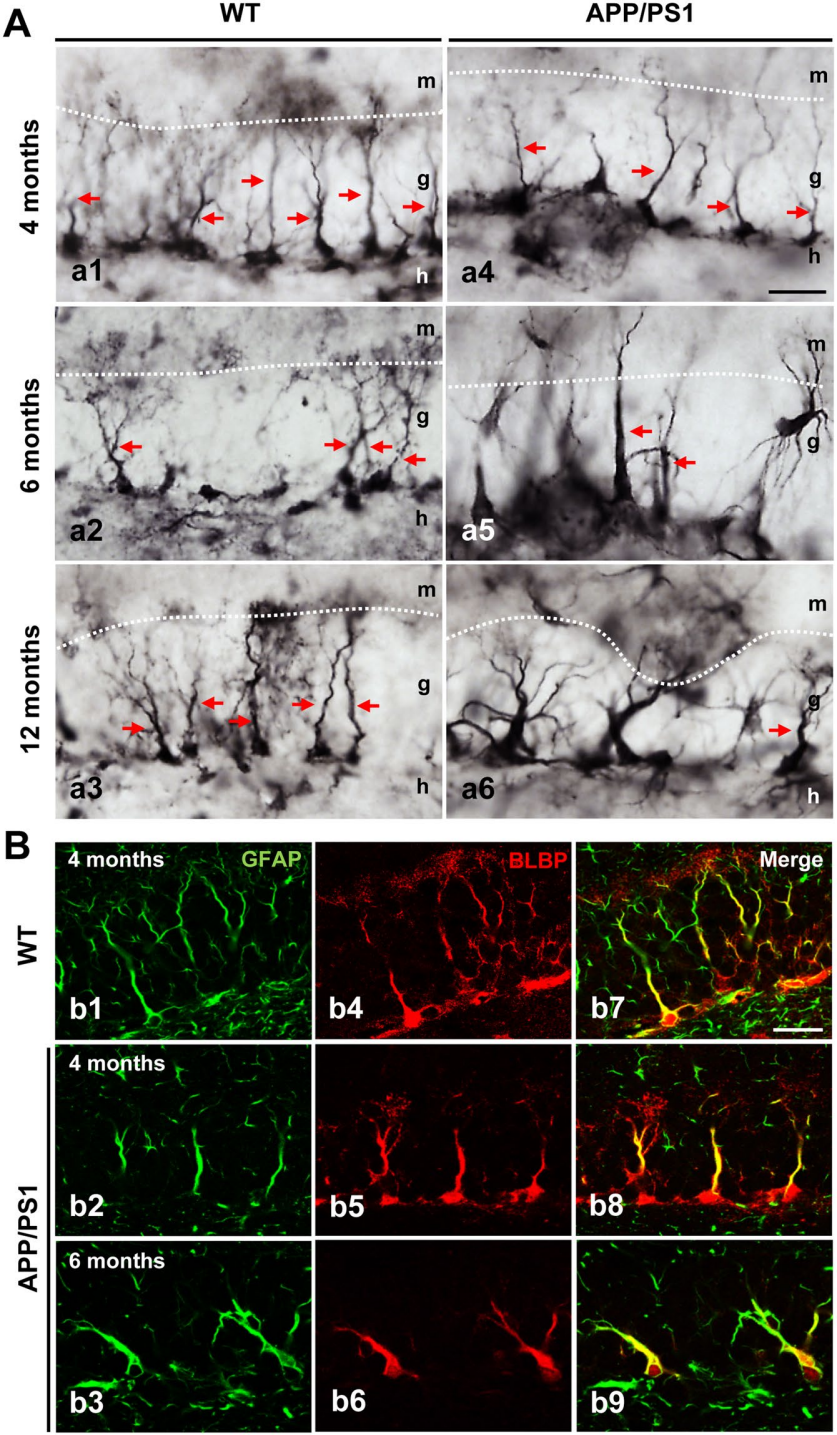
BLBP-positive stem cells in the SGZ in WT and APP/PS1 mice. (**A**) Light microscopic images of the dentate gyrus of WT (a1–a3) and APP/PS1 (a4–a6) mice immunostained for BLBP. (**B**) Double confocal images of GFAP (b1–3) and BLBP (b4–6) markers demonstrate the presence of radial glial-like stem cell type-1 progenitors in both WT and APP/PS1 mice as shown in the co-localization images (b7–9). g: granular cell layer; h: hilus; m: molecular cell layer. Scale bars: 25 μm (a1–a6 and b1–b9).

**Figure 4:**
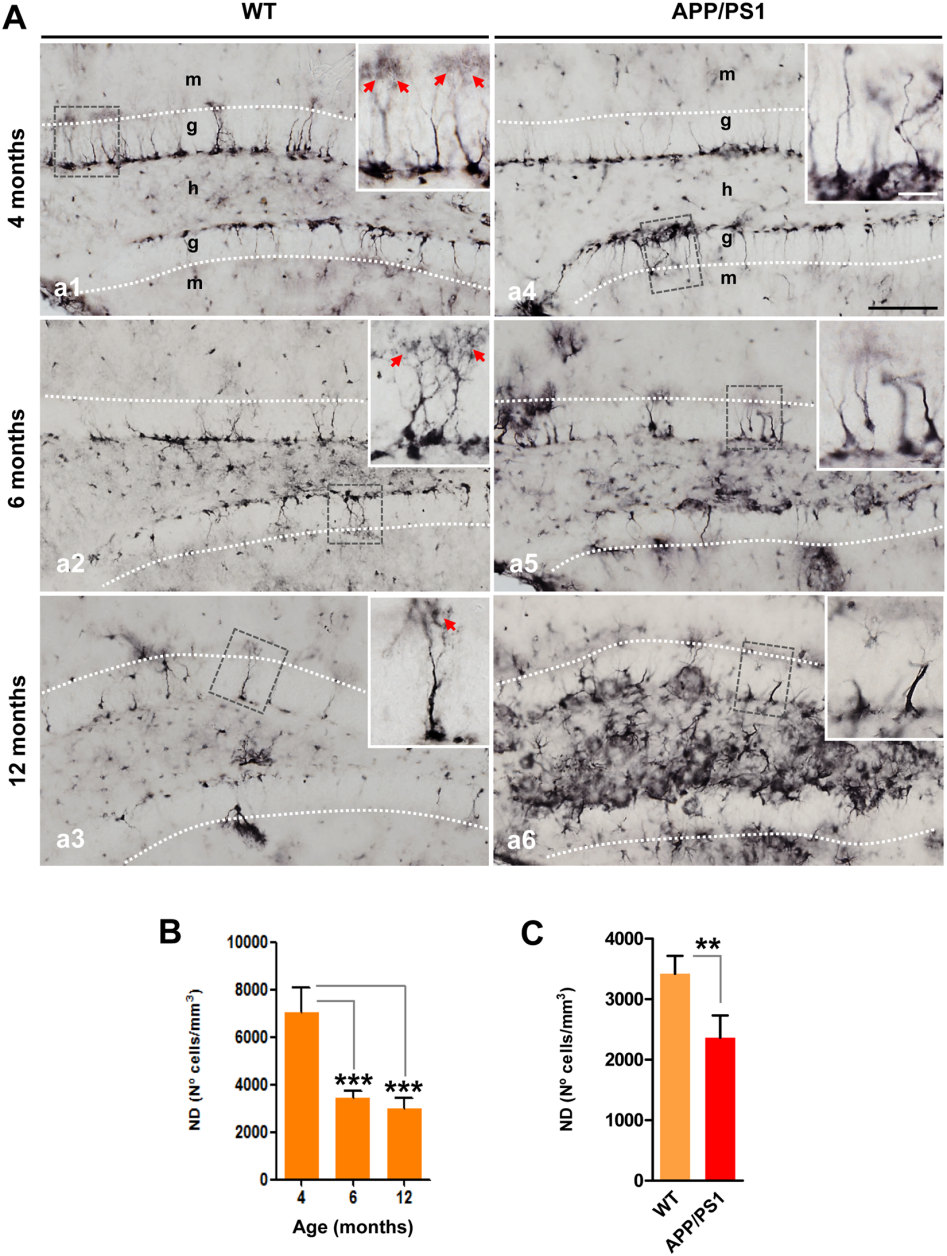
BLBP-positive stem cells are reduced in aging and in APP/PS1 mice. (**A**) Light microscopic images of the dentate gyrus of WT (a1–a3) and APP/PS1 (a4–a6) mice immunostained for BLBP. (**B**) Stereological quantification demonstrates an age-depend decrease in BLBP-positive cells (n = 4/genotype/age; ANOVA F(2,8) = 38.93, Tukey’s post hoc test, ***p < 0.001). (**C**) Stereological quantification shows a significant decrease in the number of BLBP-cells in APP/PS1 mice compared to age-matched WT mice (n = 4/age/genotype; t-test **p < 0.01). Red arrows: BLBP-positive stem cells with broccoli-like cell endings. g: granular cell layer; h: hilus; m: molecular cell layer. Scale bars: 100 μm (a1–a6), 25 μm for inserts.

Next, we seek to determine whether the reduction of cell proliferation was related to changes in progenitor cells in the SGZ of APP/PS1 mice at 6 months of age. Radial glia-like stem/precursor cells, or type-1 cells, are quiescent and pluripotent cells that can differentiate into immature neurons in the SGZ. These cells express the astrocytic markers glial fibrillary acidic protein (GFAP), nestin and brain lipid binding protein (BLBP), and can divide asymmetrically to generate intermediate progenitors (type-2 cells), which express stem cell markers such as Sox2 and neuronal lineage markers such as Prox1, NeuroD1 and DCX^32, 33^. Here, we investigated whether changes in type-1 and type-2 progenitor cells are associated with the reduction of neurogenesis in APP/PS1 mice. First, we analyzed the number of BLBP-positive cells to investigate radial glia-like stem cell type-1 progenitor cells in the SGZ^34^. Immunohistochemical analysis demonstrated the localization of multiple BLBP-positive stem cells in the SGZ of both WT and APP/PS1 mice at 4-, 6- and 12-months of age (Figs 3A and 4A). In WT and APP/PS1 mice, these stem cells showed a cell soma located at the SGZ, had an elongated main process that crossed the granular layer and displayed a terminal arborization (see Fig. 3). However, in APP/PS1 mice, BLBP-positive stem cells with thick primary processes and BLBP-positive reactive astrocytes were also observed (see Fig. 3A a4–6 and Fig. 4A a4–6). The radial glia-like type 1 cells co-expressed BLBP and GFAP as shown by confocal microscopy in the SGZ of both WT and APP/PS1 mice (Fig. 3B). Moreover, stereological quantification demonstrated an age-dependent decrease in BLBP-positive stem cells of the SGZ. In WT mice, a significant reduction of BLBP-positive stem cells at 6 months (−51.39% ± 4.23%; Tukey’s post hoc test, p < 0.001) and 12 months (−57.64% ± 6.11%; Tukey’s post hoc test, p < 0.001) compared to 4-month-old WT mice (Fig. 4A and B) was detected. Moreover, a further reduction of BLBP stem cells was observed in APP/PS1 mice at 6 months of age compared to age-matched WT mice (−30.86% ± 10.77%, t-test p < 0.01) (Fig. 4A a2 and a5, and Fig. 4C). No differences were observed at 4 months between WT and APP/PS1 mice.

**Figure 5:**
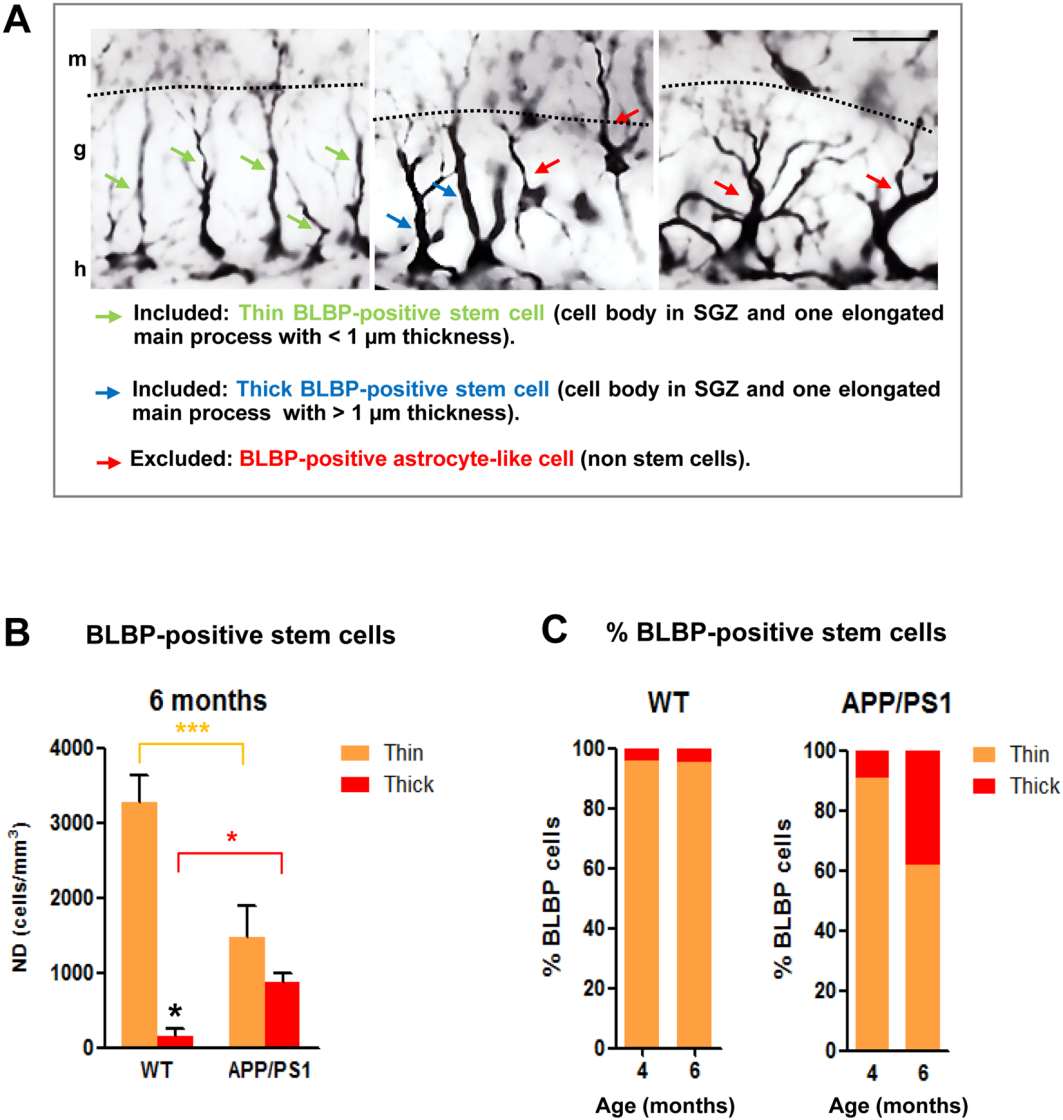
Thin and thick BLBP-positive stem cells are differentially affected in APP/PS1 mice. (**A**) Representative images of BLBP-positive cells in the dentate gyrus of 6-month-old APP/PS1 mice; radial glia-like stem cells (type-1 cells) display a thin (green arrows) or thick (blue arrows) long radial process that extends from the cell body in the SGZ and traverses the granule cell layer; BLBP-positive non-stem cells exhibit a cell body located out of SGZ and many branched processes (red arrows) resembling mature astrocytes. (**B**) Stereological quantifications show a significant decrease in the number of BLBP-stem cells extending thin apical process in the APP/PS1 mice compared to WT mice at 6 months of age. However, a significant increase in the number of BLB-stem cells with a thick process is observed in these APP/PS1 mice compared to WT mice (n = 4/genotype/age; ANOVA F(3,12) = 85.545; Tukey’s post hoc test, *p < 0.05, ***p < 0.001). (**C**) Percentage of thin and thick BLBP-positive stem cells in WT and APP/PS1 at 4- and 6-month-old. g: granular cell layer; h: hilus; m: molecular cell layer. Scale bars: 25 μm.

Herein, we also distinguished between BLBP-positive stem cells with thin or thick elongated main processes (see Fig. 5A for morphological details). Our stereological quantification (Fig. 5B and C) demonstrated a significant decrease in the thin BLBP-positive stem cells in APP/PS1 mice compared to WT mice (−55.57% ± 14.02, Tukey’s post hoc test, p < 0.001) at 6 months of age. However, a 4.5-fold increase in the thick BLBP-positive stem cells was observed in APP/PS1 mice compared to WT mice at 6 months of age (Tukey’s post hoc test p < 0.05). The proportion of thick BLBP-positive stem cells was higher in APP/PS1 mice and this number increased with age as show in Fig. 5C.

In addition, qualitative observations in semithin sections (1–1.5 μm-thick) showed a severe loss of neurogenic niches in the SGZ of the dentate gyrus in 6-month-old APP/PS1 mice compared to WT mice. In semithin sections, type-1 cells are recognized as having a triangular soma with long, single or double apical processes that radially cross the granular cell layer (GCL); however, type-2 cells displayed an irregular heterochromatic nucleus and short cytoplasmic processes extending parallel to the SGZ^35^. The quantification of morphologically identified neural precursors showed a significant reduction in the number of both type-1 (−47.31 ± 15.18%, p < 0.05 t-test) and type-2 (−69.87 ± 30.12%, p < 0.05, t-test) cells in APP/PS1 mice compared to WT (Supplemental Fig. 2A–C). Overall, these data indicate that neurogenic niches are significantly affected in 6-month-old APP/PS1 mice, and these profound alterations in stem cell type-1 progenitor cells may be responsible for the substantial decline in proliferation rate observed in APP/PS1 mice.

### Soluble Aβ affects neuronal proliferation in the APP/PS1 hippocampus

**Figure 6:**
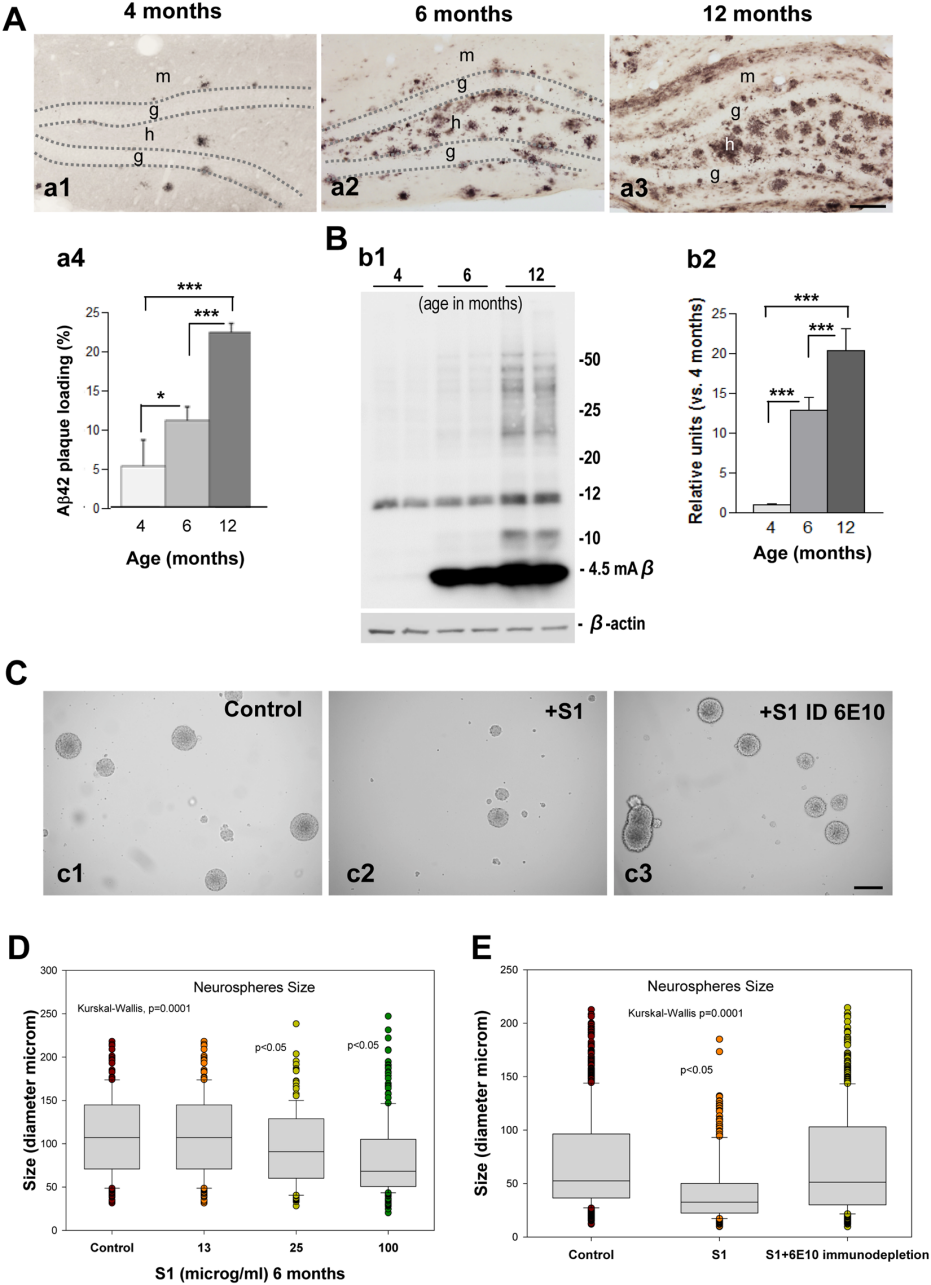
Soluble APP/PS1-derived Aβ inhibits neurospheres growth and proliferation. (**A**) Light microscopic images of the dentate gyrus of APP/PS1 mice showing an age-dependent increase in the extracellular Aβ accumulation (a1–a3); quantification of Aβ plaque loading shows a significant increase from 4 months of age up to 12 months (n = 5/age; ANOVA; F(2,8) = 54.29; p = 000.1; Tukey’s post hoc test, *p < 0.05, ***p < 0.001) (a4). (**B**) Western-blot analysis (b1) shows a significant age-dependent increase (b2) in the level of monomeric Aβ in APP/PS1 mice (ANOVA; F(2,9) = 119.3; P = 0.0001; Tukey’s post hoc test p < 0.001). (**C**) Soluble proteins (S1) from APP/PS1 mice inhibit neurospheres growth. (**D**) Aβ-immunodepleted soluble proteins from APP/PS1 mice hippocampus demonstrate that Aβ is a key molecular factor inhibiting neurospheres growth (Kruskal Wallis p = 0.0001, Dunn test p < 0.05). g: granular cell layer; h: hilus. Scale bars: 100 μm (a1–a3); 200 μm (c1–c3).

As expected from previous studies from our lab, the immunohistological analysis and plaque-load quantification demonstrated an age-dependent increase (2.1 and 4.3-fold at 6 and 12 months of age, respectively, compared to 4-month-old mice) in the extracellular Aβ deposits in the hilus of the dentate gyrus in APP/PS1 mice (Fig. 6A). In addition, Western blot analysis of hippocampal samples showed a significant age-dependent increase (12.9 and 20.3-fold at 6 and 12 months of age, respectively, compared to 4-month-old mice) in Aβ levels (Fig. 6B). Together, these data suggest that Aβ could affect neuronal proliferation in the hippocampus. To investigate whether soluble Aβ from an APP/PS1 hippocampus might affect neurogenesis in these mice, we conducted an *in vitro* neurosphere assay. The diameter of the neurosphere reflects cellular proliferative capacity. As shown in Fig. 6, the soluble Aβ present in S1 fractions derived from 6-month-old APP/PS1 hippocampal samples inhibited and/or blocked the proliferation and growth of the neurospheres (from 109.9 ± 46.38 to 83.51 ± 43.60 μm, at the maximal dose tested; Fig. 6D). To address whether soluble Aβ was the toxic agent, the neurospheres were incubated with Aβ-immunodepleted S1 samples using the antibody 6E10. Our experiment demonstrated that Aβ-immunodepleted samples did not affect neurosphere proliferation and growth (Fig. 6C and E). Therefore, our study suggests that soluble Aβ might significantly affect cell proliferation in the SGZ of APP/PS1 mice and, consequently, the formation of new neuronal cells in these mice.

### Aβ stimulates microglial proliferation in APP/PS1 mice

**Figure 7:**
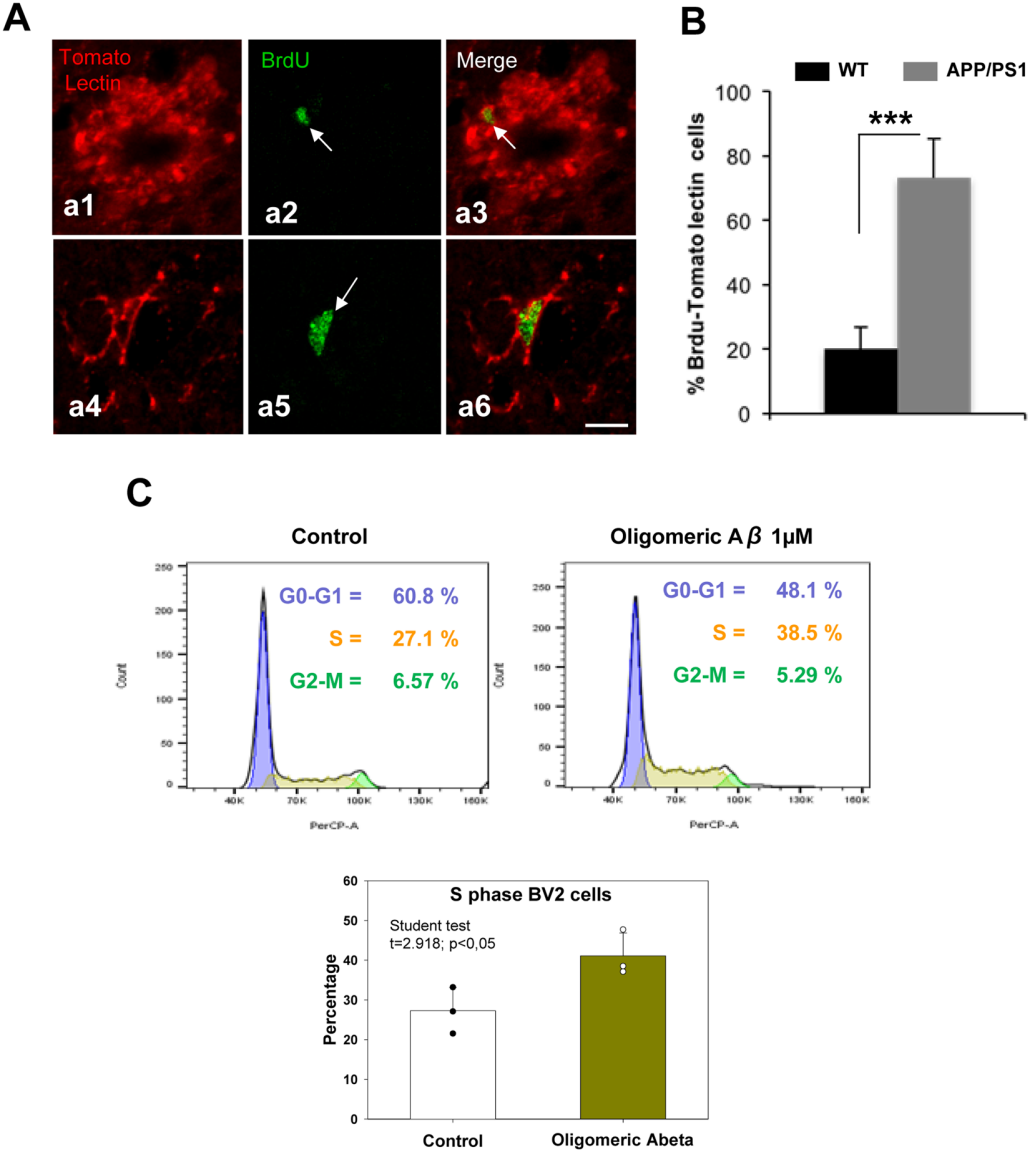
The microglial population increases in the dentate gyrus of APP/PS1 mice. (**A**) Double immunofluorescence confocal images for tomato lectin/BrdU (a1–a6) in 6-month-old APP/PS1 mice. The corresponding merged images showed that the BrdU-cells (arrows) observed in the hilus corresponded to microglial cells. (**B**) Quantitative analysis shows a significant increase of tomato lectin/BrdU double-labeled cells in APP/PS1 mice compared to WT mice (n = 6/genotype; t-test ***p < 0.001). (**C**) Bv2 proliferation assay shows an increase of proportion of microglia cells in S phase after Aβ treatment. (**D**) Proliferation index ratio shows a significant increase (showing in the graph) in Bv2 cells treatment with soluble oligomeric Aβ compared to control. Scale bars: 10 μm.

In the cell proliferation studies, we observed numerous BrdU-positive/DCX-negative cells in the hilus of the dentate gyrus of APP/PS1 mice (Fig. 2a4). To further determine the specific glial phenotype of these cells, double-immunostaining of BrdU and tomato lectin (a microglial marker) or GFAP (an astroglial marker) was performed. The confocal images and the quantitative study showed that most of the newly generated cells in the hilar region of the APP/PS1 hippocampus were microglial cells (Fig. 7A and B). The hilar microglial population increased over 3-fold (71.97 ± 9.08%; p < 0.001, t-test) in transgenic mice compared to WT mice. These results were consistent with the pro-inflammatory response that occurred in the hippocampus of these transgenic animals at these ages and coincident with the Aβ deposition. Moreover, no differences were observed in BrdU-GFAP-positive cells (Supplemental Fig. 3).

On the other hand, *in vitro* experiments demonstrated that soluble oligomeric Aβ (OC-positive) promoted cell cycle re-entry of Bv2 cells (Fig. 7C). As shown, we observed a reduction in the number of G0-G1 cells and a consequent increase in the proportion of cells in S phase. As a result, we also observed a significant increase in the “proliferation index” of Bv2 cells due to soluble Aβ (Fig. 7D). Overall, these data suggest that Aβ induces microglial proliferation.

## Discussion

The study of neurogenesis in humans and AD animal models has been a major focus of research in the past decade^3–11^; however, the role of AD pathogenesis in adult neurogenesis remains elusive. In the present study, we investigated how Aβ impairs neurogenesis in an amylogenic transgenic mouse model of AD. Our data demonstrated that neurogenesis is impaired at early ages in double transgenic APP/PS1 mice compared to WT mice. This reduction in the formation of new neuronal cells is associated with a significant decrease in the number of type-1 and type-2 progenitor cells in the SGZ of the dentate gyrus. Furthermore, we demonstrated that soluble Aβ is the key factor underlying these effects. Interestingly, our study showed that soluble Aβ forms derived from the hippocampus of APP/PS1 mice inhibited the proliferation and growth of hippocampal neurospheres, blocking the production of new neuronal cells. By contrast, we observed that the soluble Aβ stimulated microglial proliferation with no effect on astrocytes. Therefore, our study suggests that Aβ has a differential effect on proliferative cells in the CNS.

A significant decrease in SGZ proliferation, survival and differentiation has been previously shown in several AD models, such as hAPP, Tg2576 and PDAPP^36–38^. Furthermore, morphological alterations along with a dramatic reduction in dentate gyrus neural stem cells have been reported in APP/PS1 models and the 3xTg-AD model^39–42^. In the latter model, the reduction in cell proliferation was associated with the presence of Aβ plaques and significant increases in the number of Aβ-containing neurons in the hippocampus^25^. Overall, these studies are consistent with our findings in double transgenic APP/PS1 mice. In this AD model, there is a significant and early-stage (4–6 months of age) decrease in the number of DCX-positive cells in the dentate gyrus associated with a decrease in the hippocampal proliferative rate. We demonstrated that this cell-proliferation impairment is associated with a significant loss of the progenitor cells in the SGZ, as determined by stereological quantifications of radial glia-like stem cell type-1 progenitor cells (BLBP-positive) and by morphological quantification in semithin sections. The progenitor cells in the SGZ can be classified into type-1 and type-2 cells^32, 33^. Type-1 cells are astrocyte-like progenitor cells, which possess the potential capacity for self-renewal and to produce neuronal and glial cells. Type-1 progenitor cells can divide and generate intermediate precursors, defined as type-2 cells^34, 43^. These other intermediate precursor (type-2) cells remain in small clusters with a few cells in the neurogenic niches which continue to express stem/progenitor markers and progressively can differentiate into granule neurons^34, 43^. Our study demonstrates that the populations of both type-1 and -2 cells are diminished in APP/PS1 mice compared to WT mice at 6 months of age. Similar data have been reported in other transgenic animal models (i.e., APP, APPKI/PS1KI, APP23, and Tet/GSK3β) where stereological quantification using confocal images and qualitative measurements in semithin sections have shown a robust decrease in the number of progenitor cells in the SGZ^35, 38, 41, 42, 44^.

Multiple factors could be responsible for the impairment of neurogenesis in our APP/PS1 model at these early ages (4–6 months). These mice displayed a robust extracellular accumulation of Aβ in the dentate gyrus since early ages and therefore, it would be plausible that Aβ may mediate this negative effect on neurogenesis. To address this question, hippocampal neurospheres from WT mice were incubated with soluble fractions from 6-month-old APP/PS1 hippocampi. Our study clearly showed that soluble proteins from APP/PS1 mice inhibited neurosphere growth and proliferation; moreover, when these soluble samples were immunodepleted with anti-Aβ antibodies no effect on neurosphere size was detected. Thus, our data strongly supports soluble Aβ forms as the toxic agent for the formation of new neuronal cells in the hippocampus of APP/PS1 mice. Similar results from other studies support our findings, suggesting that Aβ reduces the cell viability of the neurosphere and promotes its senescent^45–48^.

Among the possible mechanisms by which Aβ impairs neurosphere proliferation, differentiation and/or migration, multiple different processes could occur in the APP/PS1 mice: the activation of formylpeptide receptor 2 (FPR2) and its downstream reactive oxygen species p38 mitogen-activated protein kinase (ROS-p38 MAPK), the activation of glycogen synthase kinase-3β (GSK-3β), or a deficiency in the nuclear factor erythroid 2-related factor (Nrf2)^45, 47, 48^. However, it is also possible that other APP-derived fragments may have a significant role in modulating neurogenesis in APP/PS1 mice^49^. Previous studies have shown that sAPPα and the APP intracellular domain (AICD) regulate neurogenesis^49^. While sAPPα has been shown to be neuroprotective and important for neurogenesis, AICD negatively modulates neurogenesis^49^. Thus, we cannot discard the idea that APP-derived fragments may also modulate neurogenesis in our APP/PS1 mice.

Despite the widely accepted idea that the formation of new neurons is impaired in AD mouse models, such as our APP/PS1 model, conflictive observations have been reported in several other transgenic mice. For example, PDGF-APPSwe,Ind mice (J20) show an increased number of BrdU-positive cells and more immature neuronal markers in the dentate gyrus than their WT counterparts^30^. Similar results are observed in the APP23 and in the double APP_K670N/M671N_/PS1_M146L_ transgenic mice^17, 28, 41^. These discrepancies could be attributed to multiple factors principally related to the transgene expression, mouse line and experimental conditions. Likewise, there are also conflicting data regarding how neurogenesis is affected in human AD cases. While some studies suggest that neurogenesis is impaired in AD^18–20^, other studies indicate that neurogenesis is stimulated by an increase in the proliferation rate and survival of new neurons^16, 17^. However, novel findings suggest that the observed increase in the cell proliferation mainly results from glial and vascular-associated changes^18, 19, 21, 22^. In this context, our results in APP/PS1 mice demonstrate that the significant increase in the number of BrdU-positive cells in the hilus of the dentate gyrus correspond to glial cells, specifically microglia. These data correlate with an important inflammatory response observed in these mice at early ages^50–52^. Using Bv2 cells, we also demonstrate that soluble Aβ oligomers stimulate microglial proliferation, which suggests that the Aβ pathology might be the causative factor inducing microglial proliferation in the dentate gyrus. In alignment with our data, several studies have shown that Aβ stimulates microglial proliferation *in vitro*, and multiple molecular mechanisms have been proposed, such as increased microglial release of TNF-α and production of hydrogen peroxide from NADPH oxidase or increased expression of macrophage colony-stimulating factor (M-CSF) through the phosphatidylinositol 3-kinase (PI3-kinase), protein kinase B (Akt) and nuclear factor κB (NF-κB) pathway^53, 54^. Moreover, oligomeric Aβ could stimulate microglial proliferation by interacting directly and/or indirectly with TREM2 (Triggering Receptor Expressed on Myeloid Cells), which is overexpressed in plaque-associated microglia^55^. In support of this idea, significant microglial proliferation has been observed in aged mice with Aβ pathology^56^, and more importantly, microglial proliferation is reduced in TREM2^−/−^ mice, which likely contributes to the reduced number of total microglia observed in older TREM2^−/−^ mice with Aβ pathology^56, 57^. On the other hand, the lack of changes in astrocyte proliferation is consistent with other reports in transgenic mice^58^ and, more relevant, in AD brains^18, 59^.

Overall, the findings from this study demonstrate important compelling evidence related to the role of Aβ in different cell populations. On one hand, Aβ impairs neurogenesis via reducing the number of progenitor cells and affecting their proliferation and differentiation into new neuronal cells. On the other hand, Aβ stimulates microglial proliferation, indicating that Aβ differentially affects the proliferative process of distinct cell types in CNS. These results provide important significant value to understanding the impact of AD in distinctive proliferative cells in CNS. Adult neurogenesis plays an important role in many aspects of hippocampus-dependent memory^60, 61^. Neurogenesis impairment, along with the synaptotoxic effect of oligomeric Aβ^62^, might be responsible for the early cognitive deficits in AD patients. The regulation of endogenous neurogenesis is an important therapeutic target for mitigating the hippocampal dysfunction associated with multiple neurodegenerative disorders, including Alzheimer’s disease.

## Methods

### Transgenic mice

The generation and characterization of APP/PS1 transgenic mice has been previously described^50–52, 63–65^. Transgenic mice (C57BL/6 background) were obtained by crossing heterozygotic Thy1-APP751SL (Swedish-K670N, M671L- and London-V717I-FAD mutations) transgenic mice with homozygotic PS1M146L transgenic mice (Charles River, France). Transgenic mice at 2, 4, 6, 9, 12 and 18 months of age were used. Age-matched non-transgenic (WT) mice of the same genetic background were analyzed as controls. Only male mice were used in this work. All animal experiments were conducted in accordance with the European Union regulations and approved by the Committee of Animal Use for Research at Malaga University.

### BrdU labelling

BrdU (Sigma-Aldrich) was dissolved in phosphate buffered saline (PBS) and administered intraperitoneally (50 mg/kg) twice a day (at 8 h intervals) for 3 consecutive days to 2- and 6-month-old APP/PS1 (n = 5/age) and WT (n = 5/age) mice.

### Tissue preparation

After deep anesthesia with sodium pentobarbital (60 mg/kg), mice were transcardially perfused with 0.1 M PBS, pH 7.4, followed by a solution of 4% paraformaldehyde (PFA), 75 mM lysine, and 10 mM sodium metaperiodate in 0.1 M phosphate buffer (PB), pH 7.4. Brains were then removed, post-fixed overnight in the same fixative at 4 °C and cryoprotected in 30% sucrose. Sections (40 μm thickness) were obtained from the coronal plane using a freezing microtome and serially collected into wells containing cold PBS and 0.02% sodium azide.

To study neurogenic niches in the dentate gyrus and identify type-1 and -2 cells in semithin sections, 6-month-old APP/PS1 and WT mice (not injected with BrdU) were intracardially perfused with 0.9% saline followed by 2% PFA and 2.5% glutaraldehyde. Next, coronal sections (200 μm), corresponding to the same hippocampal region from APP/PS1 and WT mice, were post-fixed in 2% osmium tetroxide (OsO_4_) for 2 h, rinsed, dehydrated, and embedded in Durcupan (Fluka BioChemika, Ronkonkoma, NY, USA). Serial semithin sections (1.5 μm) were cut with a diamond knife and stained with 1% toluidine blue.

### Immunohistochemistry

Serial sections from BrdU-injected control and transgenic mice were assayed simultaneously. For single immunolabeling, free-floating sections were first treated with 3% H_2_O_2_/10% methanol in PBS and with an avidin-biotin Blocking Kit (Vector Labs, Burlingame, CA, USA). The sections were incubated overnight with anti-BLBP (1:3000 dilution; Abcam, Cambridge, United Kingdom), anti-DCX polyclonal goat antibody (1:1000 dilution; Santa Cruz Biotechnology, Santa Cruz, CA, USA) or anti-6E10 (1:1500; Sigma-Aldrich, Saint Louis, USA). The tissue-bound primary antibody was detected by incubating with the corresponding biotinylated secondary antibody (1:500 dilution, Vector Labs, Burlingame, CA, USA) and then by incubating with streptavidin-conjugated horseradish peroxidase (Sigma-Aldrich) diluted 1:2000. The peroxidase reaction was visualized with 0.05% 3-3′-diaminobenzidine tetrahydrochloride (DAB, Sigma-Aldrich), 0.03% nickel ammonium sulfate and 0.01% hydrogen peroxide in PBS. Sections were then mounted on gelatin-coated slides, air dried, dehydrated in graded ethanol solutions, cleared in xylene and cover-slipped with DPX mounting medium (BDH Chemicals Ltd., England). The specificity of the immune reactions was controlled by omitting the primary antiserum.

For double immunoperoxidase BrdU/DCX labeling, free-floating sections were first treated in 2 N HCL for 30 min at 37 °C followed by 0.1 mol/L borate buffer (pH 8.5) for 15 min. Next, the free-floating sections were treated with 3% H_2_O_2_/3% methanol in PBS and with avidin-biotin Blocking Kit (Vector Labs, Burlingame, CA, USA). The sections were incubated 48 h at room temperature with rat monoclonal anti-BrdU (1:500 dilution; Serotec, Oxford, United Kingdom). The tissue-bound primary antibody was detected following peroxidase reaction with DAB including 0.03% nickel ammonium sulfate (black reaction end-product). Then, sections were incubated overnight with the second primary antibody, anti-DCX polyclonal goat antibody (1:1000 dilution; Santa Cruz Biotechnology). This second immunoperoxidase reaction was developed with DAB only (brown reaction end-product). Sections were finally mounted on gelatin-coated slides, air-dried, dehydrated in graded ethanol, cleared in xylene and cover-slipped with DPX.

For double BrdU/Tomato lectin fluorescence labeling, sections were incubated 48 h at room temperature with rat anti-BrdU (1:500 dilution; Serotec) and 60 min with tomato lectin solution (5μ/ml). BrdU/Tomato lectin double labeling was visualized with Alexa 568 donkey anti-rat (1:1000 dilution, Molecular Probes), and streptavidin-conjugated Alexa 488 (1:2000 dilution; Molecular Probes). For double GFAP/BLBP immunofluorescence labeling, sections were sequentially incubated with BLBP (1:3000 dilution, Abcam) and GFAP (1:10000 dilution; Dako, Glostrup, Denmark) followed by the corresponding Alexa 488/568 secondary antibodies. Sections were mounted onto gelatin-coated slides, cover-slipped with 0.01 M PBS containing 50% glycerin and 2.5% triethylenediamine and then examined under a confocal laser microscope (Leica SP5II).

### Cell counting

The numerical density (ND; cells/mm^3^) of the BrdU/DCX-positive cells was estimated by stereological analysis according to the optical fractionator method as we have previously described^50–52, 64^. Briefly, the immunopositive cells were visualized using an Olympus BX61 microscope (Olympus, Denmark) coupled to a DP71 digital camera (Olympus.) The New CAST-Grid Vis: 6.1.1.0 software package (Visiopharm Integrator System) generated sampling frames with a known area and directed the motorized X-Y stage (Prior ProScan, Prior Scientific Instruments, Cambridge, UK). BrdU/DCX-positive cells from 2- and 6-month-old WT and APP/PS1 mice were quantified through the entire antero-posterior extent of the hippocampus between -0.94 mm anterior and 3.64 mm posterior to Bregma coordinates. An average of 10–12 serial sections (with 280 μm between sections) per animal was analyzed (n = 5/genotype/age). This selection criteria prevented counting cells from contiguous sections. The dentate gyrus was defined using a 4X objective and the number of cells was counted using a 100X/1.35 objective. We used a counting frame of 918.43 μm^2^ with step lengths of 68.54 μm. The numerical density was estimated as follows: ND = Q/(ΣA*h), where ‘Q’ is the number of disector-counted cellular profiles, ‘ΣA’ is the area of the counting frame, and ‘h’ is the disector height of the optical disector (10 µm). For the numerical density of BLBP-immunopositive stem cells in the SGZ of the dentate gyrus, quantification was conducted according to the optical fractionator method as described above. For this study, APP/PS1 and WT mice from 4, 6 and 12 months of age were analyzed (n = 4/genotype/age; 7–9 sections per animal) using a counting frame of 22890 μm^2^ with step lengths of 213.96 μm. Only BLBP-positive cells with a subgranular position and one elongated main process were included for quantification, distinguishing between thin and thick BLBP-stem cells (prolongation thickness with less or more than 1 µm, respectively). Other events as astrocyte-like cells, positive also for BLBP, were excluded (for detail see Fig. 5). The CE value for each individual animal ranged between 0.03 and 0.08.

To quantify tomato lectin/BrdU or GFAP/BrdU double labeled cells (cells/mm^2^) in 6-month-old APP/PS1 and WT mice confocal microscopy analysis was performed on serial sections (with 280 μm between sections) from −1.58 to −3.40 mm posterior of Bregma (n = 6 animals per group and genotype). The Z-stack images were taken at 40X magnification throughout the dentate gyrus, including the granular cell layer, subgranular zone, and hilus. All double-labeled cells were imaged using the lambda-strobing function to prevent non-specific cross-excitation of fluorophores. For comparative studies, identical laser and software settings were utilized for all analyzed sections. Z-stacks were stitched into one continuous 40X representation of the entire dentate gyrus for each slice.

Type-1 and type-2 cell quantification was carried out on nine of the forty-five serial toluidine blue-stained semithin sections (one out of each five sections, with a five-section interval between stained sections) per mouse and on three mice per group. Data are reported as the mean ( ± standard deviation, SD) of the number of type-1 cells and type-2 cells per millimeter square in the DG. The length measurements of DG sections were performed with the ImageTool program^32^.

### Western blot analysis

Dissected hippocampi from 2-, 4-, 6- and 12-month-old APP/PS1 and WT mice were incubated in TriPure Isolation Reagent (Roche) to extract RNA. After that, protein pellets obtained from the TriPure Isolation Reagent- and isopropanol-mediated precipitation were resuspended in 4% SDS and 8 M urea (in 40 mM Tris-HCL, pH 7.4) and rotated overnight at room temperature to complete protein solubilization. Western blot analysis was performed as described^50^. Briefly, 5–20 μg of protein from the different samples were loaded onto a 12% Tris-Glycine-SDS-PAGE gel and transferred to nitrocellulose (Hybond-C Extra; Amersham). After blocking, membranes were incubated overnight at 4 °C in anti-DCX polyclonal goat antibody (1:1000 dilution; Santa Cruz Biotechnology) or the monoclonal 6E10 antibody (1:1500 dilution; Sigma-Aldrich) prepared in 5% non-fat milk. Membranes were then incubated in the corresponding horseradish-peroxidase-conjugated secondary antibody (Dako, Denmark) at a dilution of 1:8000. Each blot was developed using the ECL-plus detection method (Amersham) and quantified using Image-Quant Las 4000 mini gold (GE Healthcare Bio-Sciences). For normalization purposes, proteins were first estimated by the Lowry protein assay and protein loading was corrected by beta-actin. Western blot images were analyzed using the PCBAS program. In each experiment, the intensity of DCX or Aβ was corrected by the corresponding beta-actin signal. For DCX quantification, data from 2-month-old WT mice were averaged and considered as 1 relative unit. All data from WT and APP/PS1 mice were then normalized to the specific signal observed in 2-month-old WT group. For Aβ quantification, averaged data from 4-month-old APP/PS1 mice was considered as 1 relative unit.

### Bv2 proliferation assay

Bv2 microglial cells were grown (37 °C and 5% CO_2_) in RPMI 1640 supplemented with 2 mM glutamine, 10% (v/v) fetal bovine serum, plus penicillin/streptomycin (Biowest, France). For proliferation experiments, Bv2 cells were seeded onto a 24-well plate (45.000 cells/well, 12 h). The cells were serum-deprived for 8 h and then treated (12 h) with 1% (v/v) fetal bovine serum and PBS or 1 µM oligomeric Aβ(1–42) (AnaSpec; San Jose, CA, USA). Cell cycle analysis was assessed using the “PI/RNase Solution” detection kit (Immunostep; Salamanca, Spain) according to the manufacturer’s recommendations. Briefly, cells were fixed in 70% ethanol and labeled with a propidium iodide solution (PI/RNase). Cells were analyzed using a FACSCanto II flow cytometer (BD Services, San Jose, CA, USA). The *S*-*phase cell ratio* was calculated as S/(G0-G1 + S + G2-M); and the *proliferation ratio index* was calculated as (S + G2-M)/(G0/G1 + S + G2-M)^66^.

### Plaque Loading

Aβ42-immunostained dentate gyrus (DG) was observed under a Nikon Eclipse 80i microscope using a 4X objective, and images were acquired with a Nikon DS-5M high-resolution digital camera. The camera settings were adjusted at the start of the experiment and maintained for uniformity. Digital images (five sections/mouse from four different APP/PS1 mice per age group 4-, 6-, and 12-month-old) were analyzed using Visilog 6.3 analysis program (Noesis, France). Immunopositive plaque area within the hilar region of the DG (where the amyloid plaques are mainly concentrated) was identified by bright-level threshold, the level of which was maintained throughout the experiment for uniformity. The grayscale image was converted to a binary image with plaque and DG areas identified. Plaque loading was defined as the percentage of total DG area immunostained for Aβ42. The hilus area in each 4x image was manually outlined. The plaque loading (%) for each mouse was estimated and defined as (sum plaque area measured/sum DG area analyzed) × 100. The sums were taken over all slides sampled, and a single plaque burden was computed for each mouse. The mean and standard deviation (SD) of the plaque loading were determined using all the available data. Quantitative comparisons were carried out on sections processed at the same time.

### Neurosphere cultures

Briefly, neurospheres were obtained from the subventricular zone (SVZ) of WT mice (P7 post-natal). The SVZ tissue was digested in papain solution for 30 min in a 5% CO_2_ incubator. After that, neurosphere culture medium (NCM) was added to the plates with the SVZ tissue, and cells were dissociated using a glass pipette. Cells were then centrifuged for 5 min 300 × g at 4–8 °C, and the supernatant was removed. Next, cells were centrifuged for another 5 min and resuspended in 1 ml of NCM supplemented with growth factors, and then 100 cells/μl were plated in each well to perform the required experiments.

Immunodepletion experiments were conducted as previously described^52^. Briefly, 10 μg of protein from soluble S1 fractions prepared from 6-month-old APP/PS1 mice hippocampal samples (*n* = 4) were subjected to three sequential incubations (8–12 h at 4 °C) with 2 μg of 6E10 bound to Protein G-Sepharose. After Aβ immunodepletion, the S1 fractions were treated as above. As a control, the different S1 fractions were sequentially incubated with either Protein G-Sepharose or Protein A-Sepharose and tested in parallel experiments to the immunodepleted samples.

### Soluble protein extraction

The soluble fractions (S1) were obtained by ultracentrifugation of the homogenates as previously described^52, 63, 67, 68^. Briefly, hippocampal tissue samples were homogenized (using a Dounce homogenizer) in cold isotonic buffer (0.32 M sucrose, 1 mM EDTA, 1 mM EGTA; 20 mM Tris-HCl, pH 7.5; containing a cocktail of protease and phosphatase inhibitors, Sigma-Aldrich) and ultracentrifuged (Optima^TM^ MAX Preparative Ultracentrifuge, Beckman Coulter) at 120,000 × g, 4 °C, for 60 min. Immediately after centrifugation, the samples were aliquoted and stored at −81 °C until use. The protein content in the soluble fractions was determined by the Lowry protein assay.

### Statistical analysis

The data were subsequently analyzed by Student’s t-test comparison or one-way analysis of variance (ANOVA) followed by Tukey’s or Kruskal-Wallis test using GraphPad Prism software (GraphPad Prism Inc., San Diego, CA, USA). The significance was set at 95% of confidence. All values are presented as the mean ± SD.

### Data availability statement

The datasets analysed during the current study are available from the corresponding author upon reasonable request.

## Electronic supplementary material

Supplementary Material
