# Supplementary material for: Dual roles of Aβ in proliferative processes in an amyloidogenic model of Alzheimer’s disease
Source: Sci Rep. 2017 Aug 30;7:10085. doi: 10.1038/s41598-017-10353-7 (PMC5577311; doi:10.1038/s41598-017-10353-7)
Supplement: Supplementary file 1 — Supplementary Material [file 41598_2017_10353_MOESM1_ESM.docx]

# Supplementary Material

# Dual roles of Aβ in proliferative processes in an amyloidogenic model of Alzheimer’s disease.

# David Baglietto-Vargas, Elisabeth Sánchez-Mejias, Victoria Navarro, Sebastián Jimenez, Laura Trujillo-Estrada, Angela Gómez-Arboledas, Maria Sánchez-Mico, Raquel Sánchez-Varo, Marisa Vizuete, José Carlos Dávila, José Manuel García-Verdugo, Javier Vitorica and Antonia Gutierrez.

**
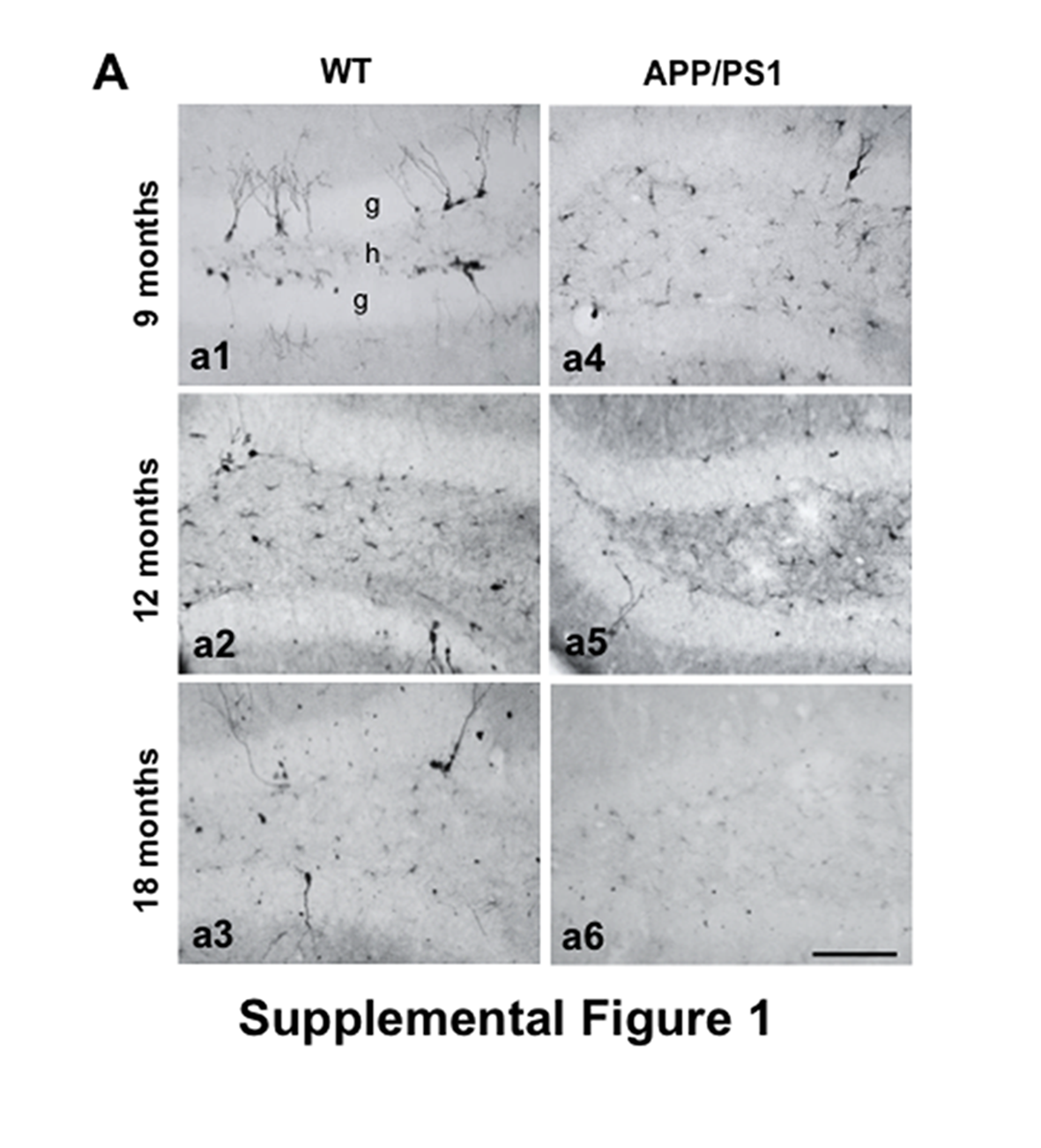
**

**Supplemental Figure 1. *DCX expression in old APP/PS1 mice hippocampus.*** A) Light microscopic images of DCX immunohistochemistry in the dentate gyrus of WT (a1-a3) and APP/PS1 (a4-a6) mice at 9, 12 and 18 months of age. g: granular cell layer; h: hilus. Scale bars: 100 μm (a1-a6).

**
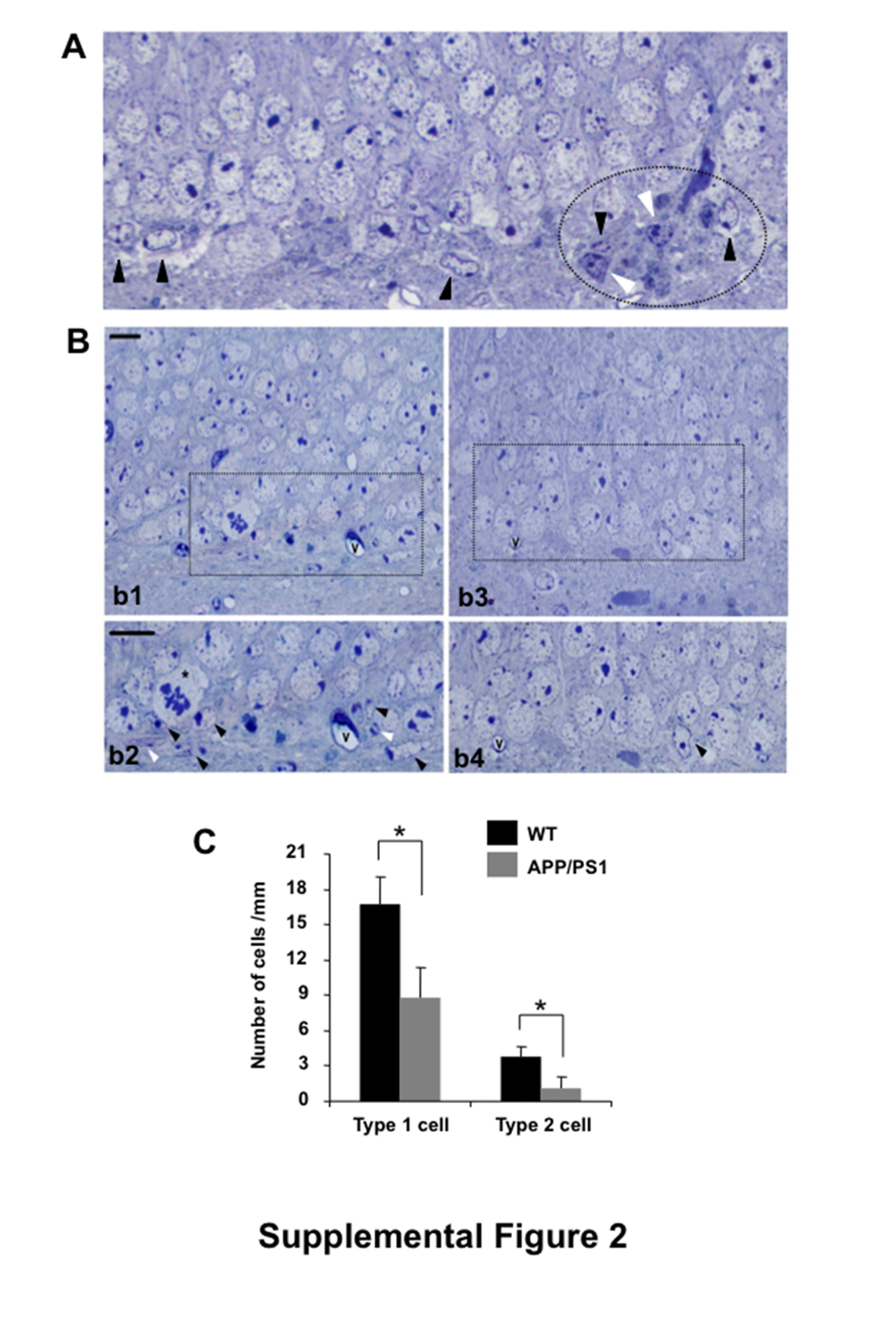
**

**Supplemental Figure 2. *Reduction of type-1 and type-2 progenitor cells in 6-month-old APP/PS1 mice hippocampus.*** A) SGZ of dentate gyrus in a toluidine blue stained semithin section (1.5 μm) of WT hippocampus showing the presence of an active proliferative niche (encircled) with radial glial-like stem cells or type-1 cells (black arrowheads) and intermediate progenitors or type-2 cells (white arrowheads). B) Light microscopic images from semithin sections (1.5 µm) stained with toluidine blue of the SGZ of WT (b1 and b2) and APP/PS1 (b3 and b4) mice at 6 months of age. The SGZ of WT animals contains abundant active niches. In APP/PS1 mice the SGZ displays a marked reduction in the number of active niches. Arrowheads same as in A; Asterisk indicates a mitotic cell. C) Quantitative analysis shows a significant reduction in the number of proliferative type-1 and type-2 cells in APP/PS1 mice compared to WT mice (n=3/genotype; t-test *p<0.05). Scale bars: 10μm (A, b1 and b3); 20μm (b2 and b4).

**
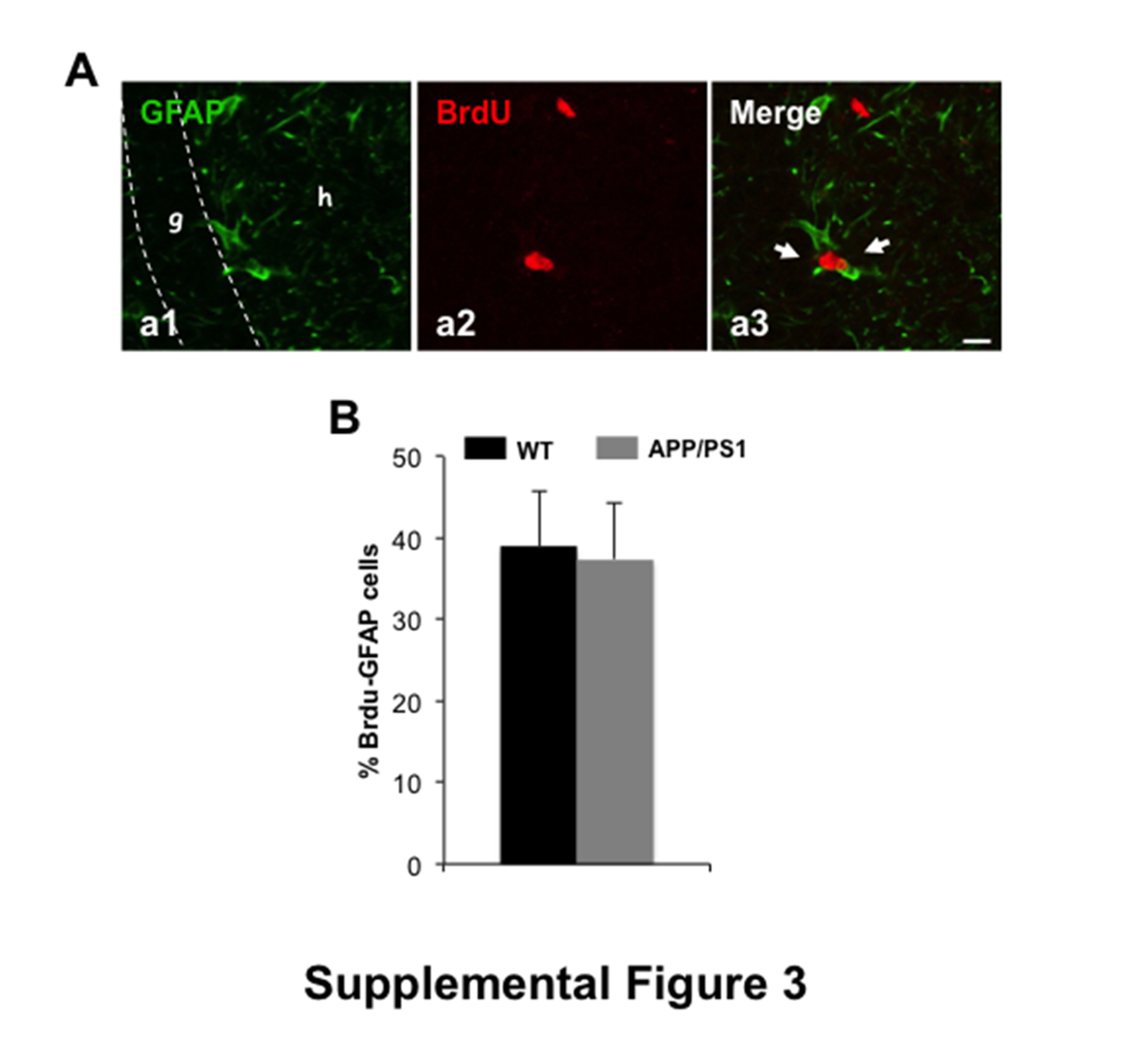
**

**Supplemental Figure 3. *Astroglia proliferation is not affected in APP/PS1 mice hippocampus.*** A) Double immunofluorescence confocal images for GFAP/BrdU (a1-a3) in APP/PS1 mice at 6 months of age. The corresponding merged image shows that the BrdU-positive cells (arrows) observed in the hilus correspond to astroglial cells. B) Quantitative analysis shows no differences in the number of GFAP/BrdU double-labeled cells in APP/PS1 mice compared to age-matched WT mice (n=6/genotype; t-test p>0.05). Scale bar: 25 μm.

**
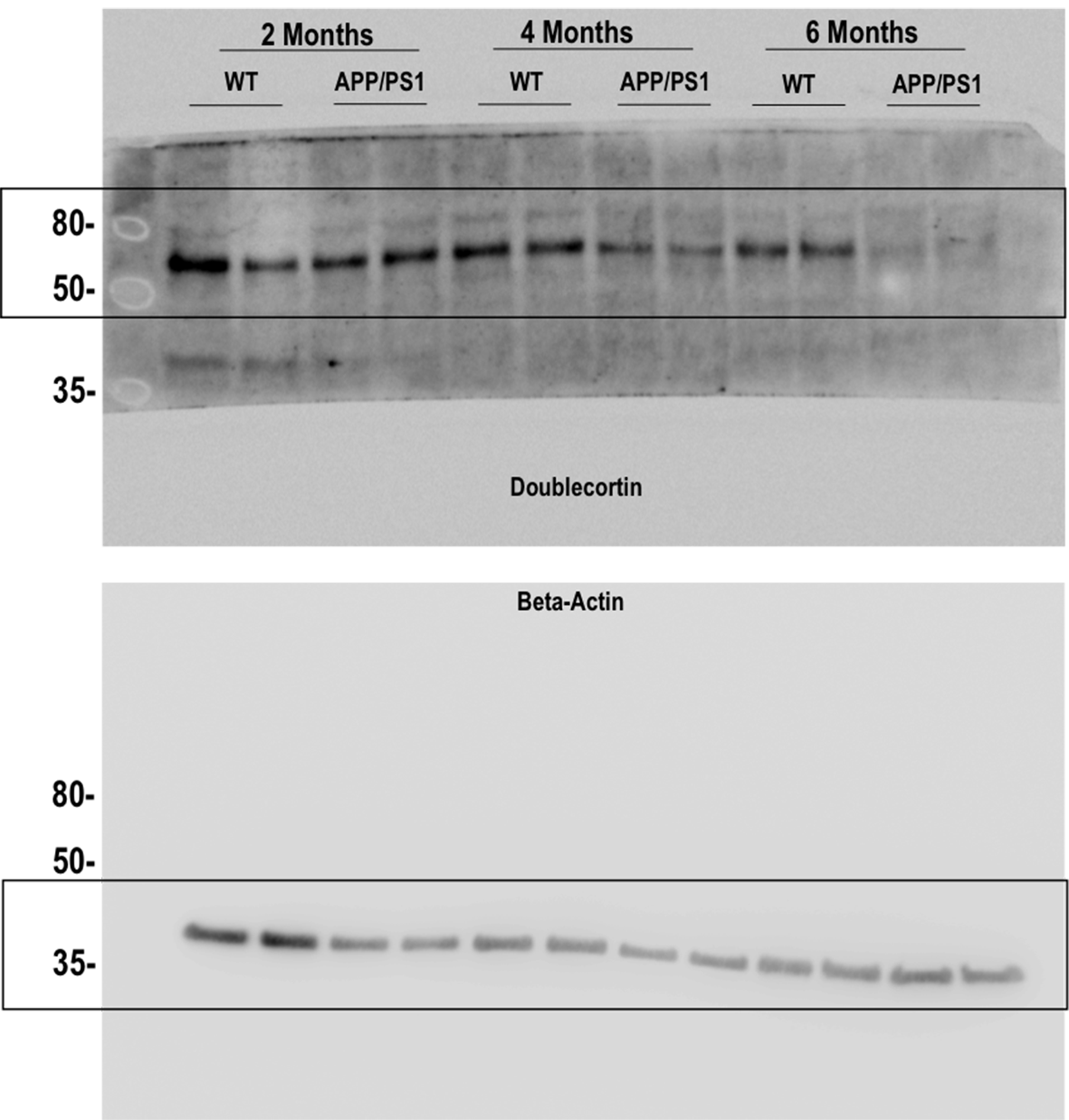
**

**Supplemental Figure. *Doublecortin levels in WT and APP/PS1 mice (full-length gel).***
